# Beneficial effects of exercise, testosterone, vitamin D, calcium and protein in older men—A randomized clinical trial

**DOI:** 10.1002/jcsm.13498

**Published:** 2024-06-18

**Authors:** Mette Midttun, Karsten Overgaard, Bo Zerahn, Maria Pedersen, Anahita Rashid, Peter Busch Østergren, Tine Kolenda Paulin, Thea Winther Pødenphanth, Linda Katharina Karlsson, Eva Rosendahl, Anne‐Mette Ragle, Anders Vinther, Rune Skovgaard Rasmussen

**Affiliations:** ^1^ Medical Department O University Hospital of Copenhagen, Herlev Hospital Herlev Denmark; ^2^ Neurological Department N University Hospital of Copenhagen, Herlev Hospital Herlev Denmark; ^3^ Department of Clinical Physiology and Nuclear Medicine University Hospital of Copenhagen, Herlev Hospital Herlev Denmark; ^4^ Department of Clinical Medicine Copenhagen University Copenhagen Denmark; ^5^ Department of Urology University Hospital of Copenhagen, Herlev Hospital Herlev Denmark; ^6^ Department of Physiotherapy and Occupational Therapy University Hospital of Copenhagen, Herlev Hospital Herlev Denmark

**Keywords:** elderly, falls, physical exercise, testosterone therapy

## Abstract

**Background:**

Due to increasing older populations worldwide, injuries, disabilities and deaths caused by falls among the elderly represent a growing human and societal problem. We aimed to improve health among men of at least 70 years of age with low‐normal to low testosterone and mobility problems by using testosterone undecanoate (TU) injections, progressive strength training, and oral supplements of vitamin D, calcium and protein.

**Methods:**

This was a single‐centre, randomized, placebo‐controlled, double‐blind trial with 148 older men with a median age of 77 (73–81) years, testosterone levels at median 8 (5–9) nmol/L (full range from 1.1 to 12.9 nmol/L) and mobility problems, recruited at University Hospital of Copenhagen, Herlev Hospital, Denmark. Participants were randomized into four arms for 20 weeks: (1) TU therapy (*n* = 37); (2) progressive resistance training with supplements of calcium, vitamin D and protein (*n* = 36); (3) both interventions combined (*n* = 36); or (4) no intervention (*n* = 39). The main outcome measure was the 30‐s chair stand test, due to test performance correlating with the risk of serious fall injuries and lower extremity muscle strength. Outcome measurements were performed at baseline and after 20 weeks.

**Results:**

After the intervention, the combination group receiving progressive resistance training, TU and supplements achieved a median score of 13 (11–15) compared to the control group at 10 (0–14) in the 30‐s chair stand test (*P* = 0.003). This median improvement of 3.0 was clinically important. Compared to the control group, participants in the combination group also increased quality of life (*P* < 0.05) and reduced both tiredness (*P* < 0.05) and leg fat (*P* < 0.05) and had higher variability in the RR interval (*P* < 0.01). The group receiving TU reduced gynoid and leg fat compared to the control group (both *P* < 0.05). Blood tests improved for several variables, especially in the combination group. There was no statistically significant increase in adverse effects from either the supplements or training.

**Conclusions:**

In men ≥70 years old with low‐normal to low testosterone and mobility problems, supplements of testosterone, calcium, vitamin D and protein combined with progressive resistance training improved 30‐s chair stand test performance, muscle strength and quality of life. Both tiredness and leg fat were reduced, and RR interval variability was increased. Significant adverse effects were not observed.

## Introduction

Among the elderly, disability and mortality after falls constitute a major health problem. Falls are the most common cause of traumatic brain injury and result in more bed days than all other accidents combined.^1^ Serious falls are associated with reduced muscle strength in the elderly, and muscle mass decreases by ~40% from 20 to 80 years of age.^2^ One week of immobilization may reduce muscle strength up to 20% and cause a loss of 1% of the maximum bone mass corresponding to the normal annual reduction.^3^ Progressive strength training during 8–12 weeks increased muscle strength by 10–45% in the elderly.^2^ The effect of strength training increased in 70‐year‐olds when supplemented with protein intake immediately after exercise.^4^ Vitamin D deficiency can lead to loss of bone mass and adversely affect the neuromuscular function,^5^ and vitamin D and calcium supplements may reduce the risk of fall in the elderly.^6^, ^7^

Testosterone levels in men decrease with age, and in ~20% of men over 60 years of age, and 50% over 80 years, serum testosterone was below 10.4 nmol/L.^8^ The normal average is ~22 nmol/L.^8^ Low testosterone levels decrease lower limb muscle function and strength, reduce bone mass, which compromise walking and balance, and increase risk of falls.^4^, ^9^ Men with testosterone levels in the lowest quartile had a 40% higher fall risk than those in the highest quartile.^9^, ^10^ Hypogonadism is also a risk factor for obesity, type 2 diabetes, atherosclerosis, myocardial infarction, chronic heart failure, dementia and erectile dysfunction.^11^, ^12^, ^13^ Men with subnormal testosterone levels had higher weight, fat mass (FM) and abdominal adipose tissue and higher glucose, insulin and triglyceride levels.^14^ Testosterone treatment of hypogonadism improved lean body mass, whole‐body protein turnover and protein synthesis, muscle mass and strength, balance, sexual function, cognition and heart rate variability (HRV) and reduced depression, major adverse cardiovascular events, type 2 diabetes and mortality.^15^ Falls are closely associated with impaired cardiac autonomic function in the elderly population, and HRV decreased with age and is further reduced in frail elderly compared to non‐frail elderly.^16^

The combination of testosterone therapy and exercise in elderly men with low‐normal to low testosterone improved quality of life, and the effect of strength training increased when supplemented with protein intake immediately after exercise and was beneficial in 70‐year‐olds.^4^ Several studies demonstrated the safety and benefits of testosterone treatment, including potential risks of prostate cancer and cardiovascular events.

In this trial, men 70 years or older with testosterone levels <13 nmol/L were randomized to testosterone injections and progressive strength training combined with protein, calcium and vitamin D supplements. Unlike previous studies, participants in this trial were older and all had mobility problems. The ability of elderly persons to perform the 30‐s chair stand test (30CST) correlates with the risk of serious fall injuries.^17^ Our main hypothesis was that the combined treatment with testosterone and training of strength supplied with calcium, vitamin D and protein would improve muscle strength, resulting in a statistically significant and clinically relevant increase in the performance of the 30CST.

## Materials and methods

### Study design and participants

This was a double‐blind, randomized, placebo‐controlled intervention trial with a 2 × 2 factorial design at the University Hospital of Copenhagen, Herlev, Denmark. The trial complied with the Declaration of Helsinki. The trial was approved by the Danish Data Protection Agency and the Regional Committee on Health Research Ethics, Protocol No. H‐16020521.

Included participants provided written consent and were consecutively recruited predominantly by adverts in local newspapers. The primary investigator provided participants with information about the trial.

Testosterone therapy should not be offered to men with total testosterone >12 nmol/L.^18^ Hypogonadism is usually associated with total testosterone levels below 10 nmol/L, although low and inadequate function is possible if total testosterone levels are between 8 and 12 nmol/L.^18^ Participants with total testosterone levels between 10 and 12.9 nmol/L were only included if they had significant and progressing mobility impairments on careful examination by an experienced medical doctor.

#### Inclusion criteria

Inclusion criteria were the following: (1) males ≥70 years of age with increased risk of falling but able to walk independently with or without use of assistive devices; (2) serum testosterone level <13 nmol/L; and (3) 30CST performance ≤12, or Timed Up and Go Test (TUG) performance ≥14 s, or if a thorough clinical evaluation supported participants' self‐reported decreasing weakness, reduced ability to walk, gait instability or repeated falls.

#### Exclusion criteria

Exclusion criteria were the following: (1) active treatment for prostate cancer or prostate‐specific antigen (PSA) >5 ng/mL if a urologist subsequently diagnosed a treatment‐requiring prostate cancer; (2) severe cardiovascular disease; (3) liver (aspartate aminotransferase [AST] greater than twice the upper normal limit) or renal insufficiency (serum creatinine >200 μmol/L); (4) epilepsy with frequent tonic–clonic seizures; (5) insulin treatment; (6) active cancer requiring chemotherapy or radiation therapy; (7) chronic disease (e.g., cirrhosis, acquired immunodeficiency syndrome [AIDS] and chronic renal failure); (8) known primary testosterone deficiency due to testicular dysgenesis, Klinefelter's syndrome (47,XXY) and other sex chromosomal abnormalities; (9) mental retardation, dementia or physical disabilities leading to inability to conduct intervention or tests or to give informed consent; and (10) contraindications for testosterone undecanoate (TU) treatment.

### Randomization and masking

**Figure 1:**
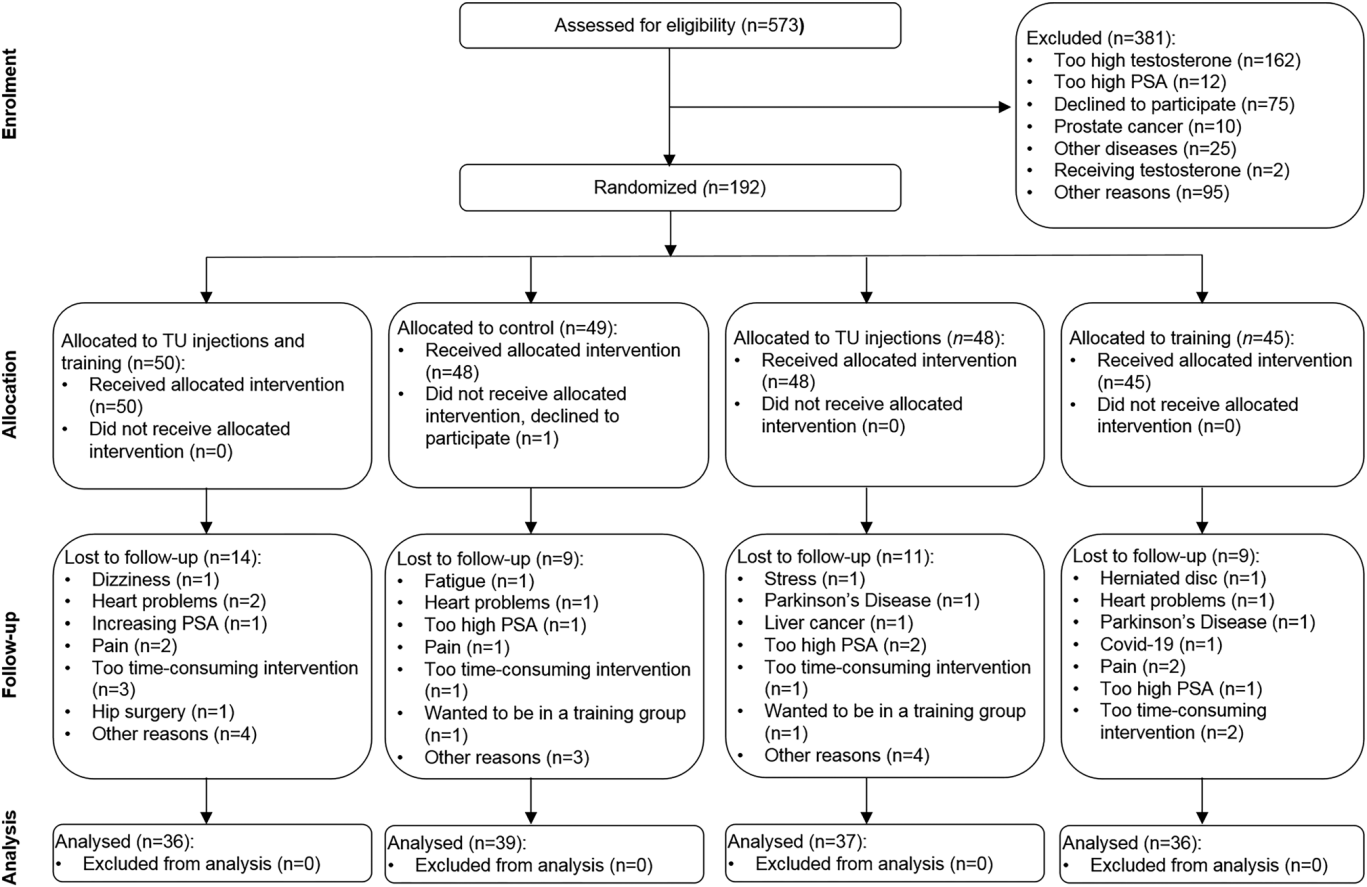
Flowchart. PSA, prostate‐specific antigen; TU, testosterone undecanoate.

Participants were randomly assigned to four groups (*Figure* *1*):

1. a combination (combo) group given 20 weeks with TU injections, and after 4 weeks, additional 16 weeks of progressive strength training with oral supplements of vitamin D, calcium and protein;
2. a control group (control) given 20 weeks with placebo injections and no physical exercise;
3. a testosterone (TU) group given 20 weeks with TU injections and no physical exercise; and
4. a training (training) group given 20 weeks with placebo injections, and after 4 weeks, additional 16 weeks of progressive strength training with oral supplements of vitamin D, calcium and protein.

To ensure equal randomization in all groups, randomization was performed so every block of 16 consecutive participants had 4 in each treatment arm in random order. Participants were randomized in two blocks: 70–79 and 80 years or older to ensure equal age distribution in all four groups. Randomization and assignments of testosterone versus placebo were performed and only known by the sponsor (Karsten Overgaard), while the primary investigator (Mette Midttun) enrolled patients.

The treatment with TU was double‐blind, while exercise with training was single‐blind because it was impossible to blind participants for exercise. Investigators blind to treatment arms enrolled participants and performed all tests. Physical training was performed by physiotherapists blind to treatment arms. All evaluations and statistics were performed blind to treatment groups.

The placebo groups had identical intramuscular injection without testosterone; the solvent contained castor oil and benzyl benzoate in an identical container (Bayer AG, Berlin, Germany). Injections were administered by medical doctors and nurses blind to treatments and with no further interaction with the participants.

### Procedures

For inclusion, the primary investigator would perform all questionnaires and the 30CST within an hour, while the physiotherapist performed the motor tests. To avoid fatigue among participants, the questionnaires and motor tests were performed on different days. As the 30CST was important for inclusion, this motor test was performed together with the questionnaires and was performed ~30 min after the beginning of the inclusion meeting. All participants were in a state of health, allowing them to participate on their own and without any need of caregivers; therefore, medical conditions were not explored. Each participant's medication was controlled by their general practitioner.

Intramuscular injections of 1000 mg TU in a 4‐mL oily solution (Nebido®, Bayer AG, Berlin, Germany) were repeated in Weeks 4 and 16. Endpoints were measured at baseline and after 20 weeks. Participants in the training groups received 20 g of protein in a bar, 14 g of protein in a drink and a daily vitamin D supplement of 38 μg in combination with 800 mg of calcium (in two doses). The 34 g of protein had positive impacts on muscle performance in older adults.^19^ Dosages of vitamin D and calcium followed clinical guidelines for elderly persons.

For safety parameters, blood parameters were collected before each injection. If 25(OH)D is <25 nmol/L, participants receive recommended treatment according to the department's usual guidelines. Before treatment with testosterone or placebo, prostate palpation and PSA measurement were performed to rule out prostate cancer. The frequency and severity of adverse events were recorded and monitored using a questionnaire for each participant.

We have previously described the training intervention in detail,^20^ and this is also available in the supporting information.

### Primary outcome

The primary endpoint was to improve the 30CST performance. The test measures the number of times a participant can rise from the seated position in 30 s and measures general strength in the lower extremities.^21^

### Secondary outcomes

Mobility‐related fatigue was assessed using the Avlund Mobility‐Tiredness Scale, which is a self‐reported six‐item scale correlating with isometric muscle strength, simple functions, increased risk of hospitalization and mortality.^S1^

The Major Depression Inventory (MDI) is a self‐reported questionnaire used to estimate depression severity and mental well‐being.^S2^

The Montreal Cognitive Assessment (MoCA) measures cognitive functions. MoCA is sensitive to mild cognitive deficits.^S3^

The quality‐of‐life EQ‐5D questionnaire was used to estimate quality of life. All scores were adjusted using Danish population norms.^S4^

The Fatigue Severity Scale (FSS) and its shortened (FSS‐7) questionnaires were used for estimating fatigue.^S5^

The modified Pelvic Organ Prolapse/Urinary Incontinence Sexual Questionnaire‐12 (PISQ‐12) provided a measurement of sexual function.^S6^

References for the above secondary endpoints using common paper tests/questionnaires are provided in the supporting information.

The Graded Cycling Test with Talk Test (GCT‐TT) was performed to measure changes in aerobic capacity.^22^

The Arm Flexion Test measured strength in the upper extremities. The number of times a participant could flex the elbow with a 3‐kg handheld weight for 30 s was counted.

TUG measured the time taken to rise from a chair, walk 3 m and return to sitting in the chair. The best of three attempts was recorded.^23^

Dual‐energy X‐ray absorptiometry (DXA) scanning measures FM, fat‐free mass (FFM) and total bone mass (BMC) and is used for regional analyses on bone mineral density (BMD) in the lumbar spine, bilateral distal forearm and proximal femur bilaterally. The same DXA scanner (GE Lunar iDXA, GE Healthcare Technologies, Madison, Wisconsin, USA) was used for all scans and the Encore software Version 17 for the subsequent analyses. Metal implants (arthroplasties, pacemakers, etc.) were counted as bone.

HRV analysis was performed on a 5‐min electrocardiogram (ECG) recording a 1000‐Hz sample rate (180 eMotion FAROS, Bittium, Finland) obtained immediately after the DXA scan, and the participants were in a supine position for 5–10 min before the ECG recording. Participants were excluded from analysis due to conditions known to influence HRV (atrial fibrillation, atrioventricular block or pacemaker). Variables expected to be affected by training and/or testosterone were selected for further statistical analysis. Variables from time‐domain analysis were mean value of RR interval (RRI) time series (mean RR), standard deviation of normal RRIs (SD RR) and square root of the mean of the squares of successive differences between normal RR (RMSSD). The Poincaré plot (a nonlinear method that plots each RRI against a previous interval) was analysed by using the minor SD1 and major SD2 axes of the fitted ellipse to the Poincaré plot. Indices were analysed by the HRV analysis software, Version 2.2 (Kubios, University of Kuopio, Finland).

### Sample size

For older men aged 70–74 years, a performance of 15 is considered normal (50th percentile) in the 30CST, whereas a performance of 11 or less is considered abnormal.^24^ We hypothesized that the control group will achieve a performance in the 30CST of 11 (64% of the 75th percentile), whereas the combination group will achieve a clinically meaningful performance improvement of 15 (88% of the 75th percentile). Using an alpha value of 0.05 and a beta value of 0.2, the needed sample size is 38 participants in each group. Due to the high age and time‐consuming intervention, up to 25% dropouts were expected, and we included 48 participants in each group.

### Statistical analysis and data collection

The data were not normally distributed. For statistical data processing, group comparisons were performed by non‐parametric tests (Kruskal–Wallis and Mann–Whitney using IBM SPSS Statistics 23). Group‐specific test score changes at baseline and after 20 weeks were evaluated using Wilcoxon's signed‐rank test for paired observations, while Spearman's rank‐order correlation test was used for ranked pairs. Fisher's exact probability test was used for categorical data comparisons that resulted from classifying data in two different ways. Lack of data was acceptable when an assessment of the primary endpoint was possible. *P* values below 0.05 were considered statistically significant. Median results are displayed as values followed by the associated 25th and 75th percentiles in brackets.

## Results

Participants were included over 4.5 years from January 2017 until September 2021. The follow‐up ended in February 2022. This trial included 192 men, and 148 completed the trial. *Figure* *1* shows the trial flowchart. Baseline results are shown in *Table* *1*, and we found no statistically significant differences for demographics, primary and secondary endpoints, blood tests, DXA and HRV. The amount of different medications was too numerous to account for, and we did not see any patterns in the medications. The lack of statistically significant differences, including blood tests and blood pressure, indicated that different medications were not a cause of bias and were equally distributed among groups. All participants were males, and as the trial was performed in Scandinavia, nearly all were white Caucasians from North Europe and lived in the urban area close to Herlev Hospital. We therefore did not include demographics for sex, race, ethnicity and location in *Table* *1*. All suffered from reduced physical activity levels. We did not thoroughly investigate socio‐economic status, but we did find a high percentage of engineers applying for trial participation (13% vs. 1.4% in the background population).

**Table 1:** Baseline characteristics

	Group			
	1 (combo, *n* = 36)	2 (control, *n* = 39)	3 (TU, *n* = 37)	4 (training, *n* = 36)
Participant characteristics and demographics				
Age (years)	77 (74–82)	76 (73–82)	77 (72–81)	76 (71–81)
Self‐reported height (cm)	178 (172–182)	180 (175–184)	178 (172–183)	178 (174–183)
Self‐reported weight (kg)	85 (73–96)	88 (80–97)	85 (76–98)	82 (77–91)
Body mass index (kg/m^2^)	27 (25–29)	27 (25–30)	27 (24–30)	26 (24–29)
Living alone (%)	28	38	30	28
Retired (%)	86	90	84	86
Never smoked tobacco (%)	28	21	27	39
Low alcohol consumption (%)	64	82	70	81
Systolic blood pressure (mmHg)	149 (136–157)	144 (133–154)	139 (131–151)	139 (132–151)
Diastolic blood pressure (mmHg)	84 (77–87)	84 (77–90)	82 (73–90)	80 (71–87)
Heart rate (b.p.m.)	68 (61–78)	68 (60–76)	73 (65–83)	66 (60–78)
Primary endpoint				
30‐s chair stand test	10 (9–13)	11 (8.5–12)	11 (8.5–12.5)	10 (7–13)
Secondary endpoints				
Timed Up and Go Test	7.6 (6.4–10.1)	8.3 (6.6–11.3)	7.7 (6.4–9.7)	8.3 (6.4–9.98)
Graded Cycling Test with Talk Test (W)	105 (75–131)	98 (75–120)	90 (75–113)	90 (75–120)
Mobility‐Tiredness Scale	0 (0–1)	0 (0–1.25)	0 (0–1)	0 (0–2)
3‐kg arm curls (repetitions in 30 s)	18 (15–20)	17 (15–20)	17 (13–21)	16 (13–19)
Montreal Cognitive Assessment	24 (22–27)	25 (22–27)	25 (24–26)	25 (23–27)
Major Depression Inventory	2 (1–5)	4 (1–8)	4 (2–9)	3 (1–7)
PISQ‐12 (average item score)	2.6 (1.9–3.1)	2.4 (2–3)	2.2 (1.2–2.8)	2.8 (2–3.3)
Fatigue Severity Scale	24 (15–43)	29 (18–46)	29 (17–45)	25 (20–45)
Fatigue Severity Scale 7	1 (1–4)	1 (1–5)	2 (1–6)	1 (1–5)
EQ‐5D	0.8 (0.78–0.86)	0.8 (0.73–0.86)	0.79 (0.74–1)	0.78 (0.65–0.8)
EQ‐5D Visual Analogue Scale (VAS)	80 (70–90)	75 (60–80)	75 (60–88)	78 (63–85)
Blood tests				
Total testosterone (nmol/L)	9.7 (7.6–12.1)	9.7 (7.4–12.6)	8.4 (6.8–12.5)	8.4 (6.7–11.6)
Free testosterone (pmol/L)	174 (114–258)	191 (151–225)	175 (131–228)	164 (112–210)
Haematocrit level (%)	42 (40–45)	43 (41–45)	42 (41–45)	43 (40–46)
Haemoglobin (mmol/L)	8.8 (8.5–9.2)	9 (8.2–9.5)	9.1 (8.5–9.4)	8.9 (8.4–9.5)
Leucocytes (× 10^9^/L)	6.7 (5.7–7.9)	6.8 (5.8–7.7)	6.3 (5.1–7.8)	7.3 (5.8–8.7)
Creatinine (μmol/L)	75 (70–90)	83 (72–95)	85 (72–101)	85 (74–97)
Glucose (mmol/L)	6 (5.6–6.5)	6.2 (5.7–7.6)	6.2 (5.4–6.9)	6 (5.5–7)
Total cholesterol (mmol/L)	4.6 (3.6–5)	4.3 (3.5–5)	4.2 (3.9–5.2)	4.2 (3.3–5.1)
HDL (mmol/L)	1.3 (1.1–1.5)	1.2 (1.1–1.6)	1.3 (1–1.6)	1.3 (1.1–1.4)
LDL (mmol/L)	2.3 (1.7–3.2)	2.2 (1.6–2.8)	2.3 (1.7–3.1)	2.4 (1.4–3.2)
VLDL (mmol/L)	0.7 (0.5–0.9)	0.6 (0.5–1)	0.6 (0.4–0.8)	0.6 (0.5–0.8)
Triglyceride (mmol/L)	1.6 (1.1–2)	1.3 (1.1–2.2)	1.4 (1.1–1.9)	1.3 (1.1–2)
Follitropin, FSH (IU/L)	9.5 (6.1–27)	9.3 (7–20)	9.4 (6.2–21)	11 (6.9–44)
Lutropin, LH (IU/L)	6.8 (3.7–13)	5.2 (4.2–7.7)	5.9 (4.2–9.2)	6.2 (4–14)
Thyrotropin, TSH (IU/L)	1.3 (1–1.8)	1.4 (0.9–2.1)	1.7 (1.1–1.9)	1.4 (0.9–1.9)
Triiodothyronine, T3 (nmol/L)	1.5 (1.4–1.9)	1.6 (1.4–1.8)	1.5 (1.2–1.7)	1.4 (1.3–1.7)
Thyroxine free, T4 (pmol/L)	15 (13–17)	16 (14–18)	16 (15–17)	15 (14–18)
PSA (ng/mL)	2 (0.9–2.8)	1.7 (0.4–2.7)	1 (0.6–2.4)	1.5 (0.7–3.9)
Vitamin D, 25(OH)D (nmol/L)	81 (63–96)	70 (63–98)	83 (67–100)	88 (62–101)
Bone densitometry (DXA)				
Height (cm)	176 (171–180)	179 (173–182)	176 (171–181)	176 (171–179)
Weight (kg)	86 (77–98)	87 (80–97)	86 (77–100)	84 (76–94)
BMD (g/cm^2^)	1.2 (1.2–1.3)	1.2 (1.2–1.4)	1.3 (1.1–1.4)	1.3 (1.2–1.3)
*T* score	0.1 (−0.5 to 0.8)	0.2 (−0.5 to 1.7)	0.9 (−0.5 to 2.0)	0.8 (−0.1 to 1.4)
*Z* score	0.9 (0.1–1.4)	0.8 (0.3–2.3)	1.2 (−0.5 to 2.6)	1.3 (0.7–1.9)
Fat mass (kg)	30 (23–35)	31 (26–36)	29 (23–34)	28 (23–33)
Fat‐free mass (kg)	55 (50–61)	56 (52–60)	56 (52–62)	54 (49–59)
BMC (kg)	2.9 (2.6–3.2)	3 (2.7–3.4)	3.1 (2.7–3.6)	3 (2.9–3.4)
Android fat (%)	42 (37–51)	44 (39–50)	41 (35–49)	43 (37–46)
Gynoid fat (%)	34 (29–36)	35 (31–37)	31 (29–35)	32 (29–36)
Legs fat mass (kg)	5.7 (4.6–6.7)	6.3 (5.2–7.8)	5.9 (4.8–7.2)	6 (4.5–7.2)
Legs fat‐free mass (kg)	17 (14–18)	16 (14–18)	17 (15–19)	15 (13–17)
Legs fat mass (%)	36 (29–39)	39 (31–50)	35 (28–43)	38 (31–43)
Heart rate variability (HRV)				
Number of participants (*N*)	21	20	20	21
Mean RR (s)	841 (761–961)	841 (753–953)	881 (711–969)	959 (865–1094)
SD RR (ms)	28 (23–37)	28 (16–37)	29 (21–45)	26 (18–57)
SD heart rate (b.p.m.)	2.4 (1.8–3.5)	2.2 (1.3–4)	3 (1.9–3.5)	1.9 (1.3–4.2)
RMSSD (ms)	29 (19–39)	26 (11–41)	25 (14–56)	25 (14–58)
SD1 (ms)	21 (14–27)	18 (7–29)	18 (10–40)	18 (10–41)
SD2 (ms)	32 (26–47)	30 (21–46)	36 (28–49)	35 (22–66)

*Note*: Median values are shown with the 25th and 75th percentiles in brackets. The Kruskal–Wallis test was used for comparisons of median values among three or more groups, and no statistically significant differences were identified. Low alcohol consumption was defined as <15 drinks per week. The reference range for LH was from 2.2 to 12.1 IU/L, and for FSH, it was <18.1 IU/L. The reference range for total testosterone was 8.4–28.7 nmol/L, and for free testosterone, it was 100–400 pmol/L (60–80 years of age) and 50–300 pmol/L (81+ years old). Due to the lower number of participants, HRV results are considered trends. DXA and HRV measures were delayed by 2–6 weeks at baseline. Abbreviations: BMC, total bone mass; BMD, bone mineral density; DXA, dual‐energy X‐ray absorptiometry; HDL, high‐density lipoprotein; LDL, low‐density lipoprotein; PISQ‐12, Pelvic Organ Prolapse/Urinary Incontinence Sexual Questionnaire‐12; PSA, prostate‐specific antigen; RMSSD, square root of the mean of the squares of successive differences between normal RR; RR, RR interval time; SD, standard deviation; TU, testosterone undecanoate; VLDL, very‐low‐density lipoprotein.

There were no patterns of exclusions. Examples of reasons for exclusions were pain due to training, fatigue, liver disease, Parkinson's disease, heart problems, too time‐consuming intervention or transportation, stress, a feeling of insufficient treatment effect, increased PSA and so forth.

The number of days between the first and second injections was 35 (31–42) days, and between the first and third injections, it was 126 (116–139) days. The groups did not differ at baseline (*Table* *1*). Groups were retested for the primary and secondary outcomes 163 (153–176) days after inclusion. At inclusion, the 148 participants had testosterone levels at 8 (5–9) nmol/L, with the full range from 1.1 to 12.9 nmol/L. More than 75% of the participants had 9 nmol/L or lower testosterone levels.

Of the 148 participants, 83% (123/148) performed ≤12 in the 30CST, 7% (10/148) had a score ≥14 in the TUG and 10% were included after a thorough clinical evaluation. This means that 17% (25/148) had higher scores than 12 in the 30CST, and these participants accounted for 7/36 in the combo group, 6/39 in the control group, 5/36 in the TU group and 7/39 in the training group.

DXA and HRV measures were delayed by 2–6 weeks at baseline and for results after the intervention.

### Comparing intervention groups with the control group at Week 20

The main results are provided in *Table* *2*. As shown in *Table* *2*, the primary endpoint 30CST improved in the combo group compared to the control group (*P* = 0.0025). Comparing the 30CST between different groups, the training group (Group 4) was not superior to the control group (Group 2), *P* = 0.76. The combo group (Group 1) was superior to the training group (Group 4), *P* = 0.007. Finally, the combo group (Group 1) was not superior to only the TU group (Group 3), *P* = 0.09. This indicated that the effect of testosterone was superior to the effect of training. In addition, 75% of participants in the combo group improved their performances in the 30CST compared to 36% in the control group, 54% in the TU group and 42% in the training group. Comparing groups for such improvements, only participants in the combo group improved more than those in the control group (*P* = 0.0011, Fisher's exact test). Only counting clinically important improvements of at least three stands, 53% improved in the combo group compared to 23% in the control group (*P* = 0.0096, Fisher's exact test), while the other groups did not differ from the control group (*P* > 0.05).

**Table 2:** Main results after 20 weeks of intervention: Primary and secondary endpoints

	Group			
	1 (combo, *n* = 36)	2 (control, *n* = 39)	3 (TU, *n* = 37)	4 (training, *n* = 36)
Primary endpoint				
30‐s chair stand test	13 (11.3–15)**	10 (0–14)	12 (6.5–14.5)	10 (0–14)
Score change since baseline	3.0 (0.3–5)**	0 (−2 to 2)	1 (−1 to 3)	0 (−5 to 2.8)
Secondary endpoints				
Timed Up and Go Test	7 (5.9–9)	7.8 (6.5–10.6)	8 (6.4–10.3)	7.7 (6.4–10.3)
Graded Cycling Test with Talk Test (W)	105 (90–120)	105 (75–120)	105 (75–105)	105 (90–120)
Mobility‐Tiredness Scale	0 (0–0)**	0 (0–1)	0 (0–1)	0 (0–1)
3‐kg arm curls (repetitions in 30 s)	17 (15–20)	17 (15–21)	17 (15–20)	17 (14–19)
Montreal Cognitive Assessment	25 (24–27)	27 (24–28)	26 (24–28)	26 (23–27)
Major Depression Inventory	2 (0–5)	4 (2–8)	3 (1–7)	4 (0–8)
PISQ‐12 (average item score)	2.7 (2–3.2)	2.6 (2–3.1)	2.5 (1.8–3)	2.4 (1.9–3.1)
Fatigue Severity Scale	21 (15–33)	27 (17–44)	21 (15–41)	23 (15–40)
Fatigue Severity Scale 7	1 (1–2)	1 (1–5)	1 (1–4)	1 (1–4)
EQ‐5D	0.86 (0.8–1)*	0.8 (0.72–0.86)	0.81 (0.74–1)	0.8 (0.72–0.86)
EQ‐5D Visual Analogue Scale (VAS)	85 (80–90)*	80 (65–90)	80 (63–86)	80 (63–86)
Bio vitals				
Systolic blood pressure (mmHg)	137 (135–150)	140 (133–147)	141 (132–153)	132 (125–156)
Diastolic blood pressure (mmHg)	81 (76–88)	83 (73–89)	84 (75–89)	75 (69–83)
Heart rate (b.p.m.)	67 (59–82)	70 (62–87)	75 (67–87)	65 (62–76)
Blood tests				
Testosterone (nmol/L)	17 (14–22)**	11 (9–14)	19 (15–27)**	10 (8.7–12)
Testosterone increase (nmol/L)	5.9 (2.4–13)**	0.7 (−0.8 to 3.8)	12 (4–14)**	0.3 (−1.4 to 4)
Haematocrit level (%)	45 (43–49)**	43 (40–44)	46 (43–49)**	43 (41–45)
Haemoglobin (mmol/L)	9.3 (8.9–9.9)*	8.9 (8.3–9.4)	9.6 (8.8–10)**	9 (8.5–9.4)
Leucocytes (× 10^9^/L)	7.1 (5.8–7.8)	6.7 (5.9–8)	6.7 (5.4–7.8)	7.1 (6.2–8.6)
Creatinine (μmol/L)	83 (72–96)	81 (73–97)	89 (80–97)	85 (76–100)
Glucose (mmol/L)	6.4 (5.2–7.4)	6.6 (6–8.1)	6.1 (5.3–7.9)	6 (5.6–7)
Total cholesterol (mmol/L)	4.2 (3.4–4.9)	4.4 (3.7–4.9)	4.1 (3.4–5)	4.3 (3.4–5.3)
HDL (mmol/L)	1.2 (1–1.4)	1.3 (1.1–1.5)	1.2 (1–1.3)	1.2 (1–1.3)
LDL (mmol/L)	2 (1.6–2.7)	2.1 (1.6–2.7)	2.3 (1.7–2.8)	2.3 (1.6–3.1)
VLDL (mmol/L)	0.6 (0.5–0.8)	0.6 (0.4–0.9)	0.6 (0.4–0.8)	0.6 (0.5–1)
Triglyceride (mmol/L)	1.5 (1.1–1.8)	1.3 (1–2.1)	1.5 (1–2)	1.5 (1.1–2.4)
Follitropin, FSH (IU/L)	1.8 (0.4–5.5)**	10 (7–20)	1.3 (0.3–4)**	10 (6.6–38)
Lutropin, LH (IU/L)	0.3 (0.1–1.6)**	5.1 (4.1–7)	0.3 (0.2–0.7)**	7 (4–17)
Thyrotropin, TSH (IU/L)	1.4 (0.9–2)	1.2 (0.8–2.1)	1.4 (1–1.9)	1.2 (0.8–2)
Triiodothyronine, T3 (nmol/L)	1.5 (1.3–1.6)	1.5 (1.3–1.6)	1.4 (1.3–1.7)	1.4 (1.4–1.7)
Thyroxine free, T4 (pmol/L)	15 (14–16)	16 (14–17)	14 (14–15)	16 (14–17)
PSA (ng/mL)	2.5 (1.2–5.2)	1.7 (0.5–2.9)	1.2 (0.8–4)	1.5 (0.7–3.2)
Vitamin D, 25(OH)D (nmol/L)	89 (75–111)**	74 (56–90)	94 (71–106)*	92 (75–114)**
Body composition (DXA)				
Height (cm)	176 (171–181)	178 (172–181)	175 (171–180)	174 (171–179)
Weight (kg)	88 (79–97)	87 (79–95)	84 (74–98)	83 (72–93)
BMD (g/cm^2^)	1.2 (1.2–1.3)	1.2 (1.1–1.4)	1.3 (1.2–1.4)	1.3 (1.2–1.4)
*T* score	0.4 (−0.5 to 0.8)	0.2 (−0.6 to 1.8)	1 (−0.3 to 2.1)	0.8 (0–1.6)
*Z* score	1.1 (0.3–1.5)	1.1 (−0.1 to 2.3)	1.4 (0.4–2.8)	1.5 (0.9–2.4)
Fat (kg)	28 (22–32)	30 (24–35)	27 (19–32)	28 (22–33)
Fat‐free mass (kg)	58 (51–62)	56 (52–59)	57 (53–63)	54 (48–58)
BMC (kg)	2.9 (2.6–3.3)	3 (2.7–3.4)	3.2 (2.8–3.6)	3 (2.9–3.4)
Android fat (%)	43 (35–48)	42 (38–48)	39 (31–47)	43 (36–47)
Gynoid fat (%)	31 (27–34)	34 (28–37)	29 (26–33)*	33 (29–37)
Legs fat mass (kg)	5.4 (4.3–6.1)	6 (4.8–7.8)	5.2 (4.3–6.9)	5.7 (4.6–7.5)
Legs fat‐free mass (kg)	17 (14–19)	16 (14–17)	16 (15–19)	15 (13–17)
Legs fat mass (%)	32 (26–37)**	39 (30–46)	33 (27–39)*	37 (31–44)
Heart rate variability (HRV)				
Number of participants (*N*)	22	20	22	25
Mean RR (s)	862 (776–988)	866 (767–937)	843 (772–913)	978 (811–1068)
SD RR (ms)	57 (30–81)**	29 (18–41)	30 (20–47)	28 (21–39)
SD heart rate (b.p.m.)	4.1 (2.2–7)**	2.1 (1.5–2.9)	2.4 (1.8–3.4)	1.9 (1.4–2.8)
RMSSD (ms)	40 (18–106)	25 (11–46)	24 (13–52)	28 (12–46)
SD1 (ms)	29 (13–75)	18 (8–33)	17 (9–37)	20 (9–33)
SD2 (ms)	58 (34–91)**	34 (24–44)	37 (25–51)	34 (26–48)

*Note*: Median values are shown with the 25 and 75 percentiles in brackets. Only variables where the Kruskal–Wallis test rejected the null hypothesis were investigated using unpaired Mann–Whitney *U* tests to identify statistically significant differences in relation to the control group. DXA and HRV measures were delayed by 2–6 weeks after the intervention. Abbreviations: BMC, total bone mass; BMD, bone mineral density; DXA, dual‐energy X‐ray absorptiometry; HDL, high‐density lipoprotein; LDL, low‐density lipoprotein; PISQ‐12, Pelvic Organ Prolapse/Urinary Incontinence Sexual Questionnaire‐12; PSA, prostate‐specific antigen; RMSSD, square root of the mean of the squares of successive differences between normal RR; RR, RR interval time; SD, standard deviation; TU, testosterone undecanoate; VLDL, very‐low‐density lipoprotein.

* *P* < 0.05 compared to the control group (Group 2).

** *P* < 0.01 compared to the control group (Group 2).

Results of the Mobility‐Tiredness Scale (*P* = 0.003) and the quality of life (EQ‐5D and the EQ‐5D Visual Analogue Scale [VAS]) (both 0.010 < *P* < 0.028) improved significantly only for the combo group compared to the control group. The combo group improved in many variables from baseline to 20 weeks, including testosterone blood levels. Only the groups receiving TU reduced leg fat compared to the control group.

The combo group had a significantly higher HRV in RRI and SD2 (long‐axis Pointcaré plot) compared to the control group.

### Isolating the effect of testosterone therapy: Combo versus training and TU versus control

The only difference between the combo and training groups was the testosterone therapy. Comparing these groups may isolate the effect of the testosterone therapy, and at baseline, the combo group only differed from the training group by having a higher EQ‐5D score (*P* = 0.03) but not for the EQ‐5D VAS (*P* = 0.22). After the intervention, the combo group achieved better results for the 30CST (*P* < 0.01), for the Mobility‐Tiredness Scale (*P* = 0.004) and for quality of life measured by both the EQ‐5D (*P* = 0.002) and the EQ‐5D VAS (*P* = 0.009).

For DXA results, the combo group had a lower BMD at baseline but not after the intervention. The combo group achieved more fat‐free mass for the legs in kilograms (*P* < 0.05) and reduced the percentage of FM for the legs (*P* < 0.01). For HRV results, at baseline, the combo group had a lower mean RR (*P* = 0.04); after the intervention, this difference was no longer found, but SD RR (*P* = 0.03), SD HR (*P* = 0.003) and SD2 (*P* = 0.03) all increased in the combo group. The training group achieved lower diastolic blood pressure than the combo group (*P* = 0.03).

Focusing on the TU and control groups, we found no statistically significant changes for the primary and secondary outcomes, apart from DXA showing decreased gynoid fat in the TU group (*P* = 0.02).

Specific changes for each group are provided in *Table* *2*.

### Compliance

Median training compliance ranged from 83% to 84% in the training groups. Information about compliance is available in the supporting information.

### Adverse events

Reported adverse events are shown in *Table* *3*. Six participants in the combo group, three participants in the training group and two participants in both the control and TU groups had elevated PSA. These participants were evaluated by a urologist, but none of these evaluations resulted in exclusion from the trial.

**Table 3:** Adverse events

	Group			
	1 (combo)	2 (control)	3 (TU)	4 (training)
Pain at site of injection	8	10	4	11
Bruising at injection site	1	0	0	0
Falls	2	0	3	1
Rib fracture	0	0	0	1
Broken ankle	0	0	0	1
Back pain	0	0	0	1
Knee pain	0	1	1	0
Thigh pain	0	0	1	0
Soreness in nipples	0	1	0	1
Screening of elevated PSA by urologist	6	2	2	3
Weight gain	2	0	0	0
Weight loss	1	0	1	1
Increased sexual appetite	2	0	0	0
Increase in temperament	1	0	0	0
Depression	0	1	0	0
Acne (face and groin)	0	0	1	0
Dry skin	0	1	0	0
Headache	0	1	0	0
Atrial fibrillation	0	1	2	0
Heart hypertrophy	0	0	0	1
Heart failure (non‐lethal)	0	0	1	0
Acute myocardial infarction (non‐lethal)	0	1	1	0
Haemorrhagic stroke (non‐lethal)	1	0	1	0
Change in sweat odour	0	0	1	0
Intestinal bleeding	0	0	0	1
Increased flow of urine	0	0	1	0
Elevated liver enzymes (high alcohol intake)	1	0	0	0
Elevated liver enzymes (low/no alcohol intake)	0	0	1	0
Liver pathology—presumably cancer	0	0	1	0
Red rash on thorax (penicillin allergy?)	1	0	0	0
Urinary tract infection	1	0	0	0
Diarrhoea	0	0	1	0
Pneumonia	0	0	1	0
Epidermitis	1	0	0	0
Fainting	0	0	0	1
Femoral fracture after a fall	1	0	0	0
Dizziness	1	0	0	0
Hypercalcaemia	0	0	0	1
Stress due to the participation	0	0	1	1
Total number of reported adverse events	30	19	25	25

*Note*: Different events were reported, along with the associated number of participants. Adverse events were rare, appeared randomly and mostly included pain at the site of injection. No statistically significant differences were observed. Abbreviations: PSA, prostate‐specific antigen; TU, testosterone undecanoate.

### Within‐group comparisons: Changes from inclusion to Week 20

Comparing how participants within a group changed performance from inclusion to Week 20 revealed intra‐group changes undetected when directly comparing groups. These results are considered exploratory trends and are provided as *Tables* *S1*–*S3*.

## Discussion

In men ≥70 years old with mobility impairments and low‐normal to low testosterone, supplements of testosterone, calcium, vitamin D and protein, in combination with progressive muscle resistance training for 20 weeks, improved muscle strength and reduced leg fat, increased FFM and improved both quality of life and HRV. No serious side effects were observed.

Compared to the control group, we could not show that this combined treatment improved cognition, fatigue levels, sexual functions or mood, but all participants had normal levels of cognitive skills, fatigue and no signs of depression at inclusion.

Testosterone supplements have been suspected of contributing to cardiovascular disorders, but in a recent meta‐analysis, no correlation between testosterone supplements and cardiovascular disorders was found in more than 100 000 participants.^25^ In contrast, testosterone deficiency is a known risk factor for atherosclerosis, myocardial infarction and chronic heart failure,^8^ and the overall health of participants receiving testosterone therapy combined with physical strength training may be improved. Directly compared to the control group, none of the intervention groups differed in cholesterol levels.

The only intervention difference between the combo and training groups and between the TU and control groups was testosterone therapy. Comparing the TU and control groups, the only difference we found was a decrease in gynoid fat in the TU group. Comparing the combo and training groups, the combo group achieved statistically significant improvements for 30CST, the Mobility‐Tiredness Scale and quality of life. Fat‐free leg mass increased, and the percentage of FM decreased, in the combo groups compared to the training group. For HRV results, the combo group increased SD RR, SD HR and SD2, while the training group achieved lower diastolic blood pressure. Our results indicate that testosterone treatment has limited or no effect unless combined with progressive muscle resistance training.

Of the 192 men participating, 148 completed the trial, which may be considered an excellent retention rate and a strength of the trial. As a limitation, this study was focused on older men with mobility impairments who were able to walk independently, which may have increased the retention rate by excluding patients with more severe needs for rehabilitation and treatment. With 36–39 participants in each group, this is a phase II trial. For the sample size, after 20 weeks of intervention, we expected the control group to have a performance of 11 in the 30CST, while a performance of 15 was expected for the combo group, for a statistically significant difference at the 0.05 level—corresponding to alpha set to 0.05. Instead, the control group achieved a median score of 10 in the 30CST, and the combo group achieved 13. This change of 3 was less than the expected change of 4, but the change of 3 resulted in a *P* value much lower than 0.05, namely, of only 0.0025. A positive change of 2.6 in the 30CST is considered a ‘major clinically important improvement’ based on measurements and feedback from patients.^26^ When starting out to conduct a trial, the strength and efficacy of a treatment are unknown. While we failed to achieve an improvement of 4 in the 30CST, we achieved a major clinically important improvement of 3.0, and the *P* value for this improvement was much stronger than we originally anticipated. Other investigators also found benefits of combining resistance training with testosterone therapy, and this combination was found to up‐regulate both myogenic gene programming and myocellular translational capacity, causing higher protein turnover and accretion.^27^

Seventeen per cent (25/148) had higher scores than 12 in the 30CST and were equally distributed with 5–7 per group. Due to the use of non‐parametric statistics, these outliers had a limited effect on any of the results. The outliers were still able to improve their results from 15 to 18 in the 30CST, and we found no signs of any ceiling effect.

The ability of elderly persons to perform the 30CST correlates with the risk of serious fall injuries and was chosen as the primary endpoint of this trial.^17^ Other investigators have found that an increased number of falls during a 1‐year period among elderly were directly associated with reduced performance in the 30CST and that the 30CST offered a method to screen for fall risk in older adults in long‐term care.^17^ Similarly, in a recent prospective cohort study, investigators found that the 30CST could be used to predict the risk of falls among older adults with excellent accuracy.^28^ The 30CST predicted falls among the elderly, but the two aforementioned studies used a modified version of the 30CST by allowing participants to use their upper extremities and armrests. The unmodified 30CST does not allow for the use of upper extremities, and any use of upper extremities and armrests is scored as a 0. We did not modify the 30CST and recorded modified performances according to normal guidelines. Being unable to perform the test indicates severe performance reductions, which may become hidden by allowing the use of upper extremities. For the 30CST, we found a median difference of 3.0 only for the combo group, while other groups improved from 0 to 1. Other investigators found that an increase of 2.6 or more in the 30CST was equal to a ‘major clinically important improvement’ based on feedback from patients.^26^ Only the combo group experienced a clinically meaningful difference. This clinically important improvement was observed after 4 months of training, and further improvements may develop if the intervention lasts 8 or 12 months.

Our interval between the initial injections was ~5 weeks, close to the normal recommendation of a 6‐week interval. We did not observe any clinically meaningful adverse effects due to this schedule.

Improved sexual function, mood, muscle power and body composition have been found in hypogonadal men treated with testosterone.^29^ We could not find evidence of changed sexual functions, but our participants often reported little interest in sexual activities or did not have a partner.

Bioavailable serum testosterone has been positively associated with improvement of sustained attention, processing speed and working memory in older men.^30^ A decline in androgen status has been associated with a cognitive decline and a higher incidence of dementia.^12^, ^13^ Interestingly, we did not find cognitive differences between groups in the MoCA. Our intervention, limited to 20 weeks, may have been insufficient to show any impact on cognition; also, our groups at baseline contained older men without signs of cognitive decline. Elaborate neuropsychological testing may have been needed to detect subtle differences between groups, and with elaborate testing, testosterone increased cognitive functions in older men with obesity and hypogonadism.^31^

It may be noted that the control group, compared to their baseline results, improved scores for both the MoCA and EQ‐5D, and these statistically significant changes were probably due to a placebo effect.

Qualitatively, investigators experienced that participants were generally satisfied with the training, even if training three times a week was a burden to some. The social aspects of being together with other men were important. After training, the men discussed different subjects while consuming their protein supplements. Such social aspects may have improved quality of life even in the control group.

The intervention lasted 20 weeks, and this limited time combined with physical exercises three times a week may have been insufficient to provide substantial physical changes in the training group. Increasing weekly training to more than three times may reduce motivation in many of the elderly, and we tried to establish a schedule for physical training, which was feasible for most elderly.

HRV is a reliable tool to diagnose autonomic impairments, a parameter known to be associated with the risk of falling. HRV declines with age and is associated with testosterone levels.^32^ Our trial indicates that the combination of supplements and training can improve HRV in elderly men with mobility problems, whereas training alone did not reveal any statistically significant effect. DXA and HRV measurements were delayed by 2–6 weeks for baseline and Week 20 results, and especially results at Week 20, which usually were closer to Week 24, may have been impacted by this delay.

Comparing the combo group to the control group, we found no statistically significant differences between PSA levels. In a recent meta‐analysis including 27 placebo‐controlled randomized controlled trials (RCTs), no evidence was found of increased PSA levels following testosterone therapy for 1 year, and the meta‐analysis found no evidence of increased risk of prostate cancer.^33^ In another study using 3‐year follow‐up in 1000 patients after testosterone therapy, there was no evidence of increased risk of prostate cancer.^34^

Men (50–70 years old) with low‐normal serum testosterone concentrations receiving 12 weeks of exercise benefitted more from training alone than testosterone treatment alone.^35^ Compared to our study, these men were younger, and different motor tests like leg press were used. For total lean mass and leg press, the group receiving testosterone and exercise achieved the most statistically significant improvements, and our results for the combo group were similar. We failed to find similar benefits from training alone, and another difference was that our group with testosterone alone improved for the DXA results. Both studies used smaller groups of <40 participants, and larger group sizes may be needed for clearer results. Compliance also seemed better among the 50‐ to 70‐year‐olds and may be important to improve among the elderly.

In another study, physical function was not improved in older men with low‐normal testosterone levels after 52 weeks of testosterone therapy with or without progressive resistance training, although especially their combination group achieved greater improvements in body composition than either intervention alone.^36^ In that study, transdermal testosterone gel was used, while we used intramuscular injections. Studies documenting substantial effects of testosterone typically employed intramuscular injections, while lower absorption with transdermal gel administration was associated with more limited myotrophic effects.^37^, ^38^ Compliance may also be reduced for gel administrations.

## Conclusions

In elderly men with low‐normal to low testosterone and mobility problems, supplements of testosterone, calcium, protein and vitamin D combined with progressive resistance training for 20 weeks improved muscle strength, quality of life and HRV and reduced leg fat. Only the combination of the supplements and training provided a major clinically important improvement. Our findings should be reproduced by others focusing on the individual effects of these supplements and resistance training before we can generally recommend prescribing these supplements and progressive muscle resistance training. To better understand the intervention's effects on different subgroups, the inclusion of a broader range of participants with varying degrees of mobility problems may be a focus in larger and longer term future investigations, also including different populations.

## Conflict of interest statement

All authors declare no conflicts of interest. Nebido and placebo were provided free of charge by Bayer AG, Berlin, Germany. No payments were received by Bayer, and Bayer did not influence the planning of the study, the data collection or the writing of the manuscript. For calcium and protein supplements, no payments were received, and the producers did not influence the planning of the study, the data collection or the writing of the manuscript.

## Funding

The project was funded by the Velux Foundation (Velux Stiftung), Foundation Juchum, Helsefonden, the Aase and Ejnar Danielsen Foundation (Aase og Ejnar Danielsens Fond), Beckett Foundation, Marie and Børge Krogh's Foundation, and Any and Richard Sperling's Foundation.

## Supporting information


**Table S1.** Endpoints with statistically significant changes within a group from baseline to week 20.
**Table S2.** Blood tests with statistically significant changes within a group from baseline to week 20.
**Table S3.** Bone Densitometry (DXA) with statistically significant changes within a group from baseline to week 20.
