# Supplementary material for: Beneficial effects of exercise, testosterone, vitamin D, calcium and protein in older men—A randomized clinical trial
Source: J Cachexia Sarcopenia Muscle. 2024 Jun 18;15(4):1451–62. doi: 10.1002/jcsm.13498 (PMC11294024; doi:10.1002/jcsm.13498)
Supplement: Supplementary file 1 — Table S1. Endpoints with statistically significant changes within a group from baseline to week 20. Table S2. Blood tests with statistically significant changes within a group from baseline to week 20. Table S3. Bone Densitometry (DXA) with statistically significant changes within a group from baseline to week 20. [file JCSM-15-1451-s001.docx]

***Training intervention***

Starting 4 weeks after the first injection and continuing for 16 weeks, participants in the training groups received training for one hour, three times weekly, led by two physiotherapists in teams of about 10 participants (reduced to 5 during the Covid-19 pandemic). The training took place in the rehabilitation facilities at the hospital, where appropriate strength training equipment (Technogym, Gambettola, Italy) and stationary bikes (Monark model 828E and 927E, Vansbro, Sweden) were used.

Participants started with a 10-min self-paced warm-up on a stationary bike followed by approximately 45 min of progressive resistance training on machines (Technogym, Element series) including the following specific machines: leg press, leg curl (hamstrings), leg extension (quadriceps), abdominal crunch, lower back, low row, and chest press. The machines were adjusted to fit the individual participants aiming for full range of motion while accommodating any individual needs of the participants. To induce adequate muscular fatigue and thus an adequate training stimulus, all sets of one exercise were completed with a 1- to 2-min rest in between sets before moving on to the next exercise. The intensity of the exercises was progressed by increasing the weight lifted and the number of sets performed while decreasing the number of repetitions in each set during the 16-week training period. The American College of Sports Medicine (ACSM) guidelines for progression were used for guidance while carefully accommodating participant feedback and special needs and challenges of the individual participants. A slow progression beginning with 2×15 repetitions of each exercise with low load for the first 2 sessions was used to give the often relatively weak patients time to get familiarized with the machines, learn the correct execution of each exercise, and adapt to the training without experiencing excessive muscle soreness. The initial loading of each exercise was decided by the supervising physiotherapist in close cooperation with each individual participant. To ensure a proper progression in the training load in accordance with the program, the weight lifted in each exercise was continuously adjusted throughout the training period by the supervising physiotherapist. If a participant could perform significantly more (2-3 repetitions) than the planned number of repetitions in each set of a particular exercise, the loading was increased to reach the desired repetition maximum (RM) - that is, the number of repetitions that can be performed with proper technique before repetition failure. If one or more training sessions were missed, the loading was adjusted by the supervising physiotherapist aiming for the number of repetitions in each set and the corresponding RM for the session.

***Compliance***

Due to the age and weakened strength of our participants, we tried to make participation as effortless as possible, because the participants had to meet for training 3 times a week in 16 weeks, and many lived several miles from the training facilities. We therefore did not ask participants to keep a logbook for daily calcium, vitamin D and protein intake. Our participants were mentally sound and highly motivated for participation, and when they met with the nurses for injections in weeks 4, 10, and 16, the primary investigator thoroughly asked each participant about compliance – and at the follow-up test at week 20. We did not experience issues indicating low compliance, instead the primary investigator was often contacted by participants when close to needing more calcium and d-vitamin. Protein intake was monitored by the physiotherapists after each training session, and even though a few participants mentioned it could be a challenge finding appetite for a protein bar and a protein drink, all participants consumed this diet. Protein bars had no added sugar and popular flavors were “Chocolate Banana”, “Cookies & White Chocolate”, “Hazelnuts & Chocolate” and “Cashews & Caramel”. Most participants enjoyed the bars and the protein drink.

Each participant in the Combo and Training groups received a maximum of 48 hours of training. Participants in the Combo group completed 41 (37–45) hours corresponding to 84 (77–94) %, while the Training group completed 40 (27–45) hours corresponding to 83 (56–94) %. There were no statistically significant differences for training compliance.

Correlations between compliance (number of training hours completed) and endpoints revealed that the Combo group reduced depressive symptoms for the MDI scores (Spearman R= -0.43, p=0.01) and increased quality of life in the EQ5D Visual Analogue Scale (Spearman R=0.54, p=0.001), but not for other endpoints. There was no significant correlation for compliance and endpoints for the Training group. We tried to pool all participants receiving training into one large group to better see any effect of training, this resulted in a group of 72 participants all receiving training. For this group, the number of training hours correlated with pulse reduction (Spearman R= -0.25, p=0.049), increased quality of life for the EQ5D Visual Analogue Scale (Spearman R=0.35, p=0.003) and reduced depressive symptoms for MDI scores (Spearman R= -0.27, p=0.026).

Participants not able to participate in the training interventions were excluded. Participants excluded due to severe compliance problems are listed in Figure 1 (Flow Chart) under the category “lost to follow-up”. Lack of compliance was especially found among participants having pain, feeling too much fatigue or finding that the intervention was too time-consuming. The number of excluded “lost to follow-up” participant was highest in the Combo group (28%), and lowest in the Control (19%) and Training (20%) groups, but there were no statistically significant differences between groups (Fisher Exact Test, all p>0.05).

**Table S1**

**Endpoints with statistically significant changes within a group from baseline to week 20**

|  | **Baseline** | **Week 20** | **p-value** |
| --- | --- | --- | --- |
| **30-Second Chair Stand Test** |  |  |  |
| Group 1 (Combo) | 10 (9 – 13) | 13 (11.3 – 15) | **0.0003** |
| Group 2 (Control) | 11 (8.5 – 12) | 10 (0 – 14) | 0.26 |
| Group 3 (TU) | 11 (8.5 – 12.5) | 12 (6.5 – 14.5) | 0.29 |
| Group 4 (Training) | 10 (7 – 13) | 10 (0 – 14) | 0.42 |
| **Timed Up And Go** |  |  |  |
| Group 1 (Combo) | 7.6 (6.4 – 10.1) | 7 (5.9 – 9) | **0.0013** |
| Group 2 (Control) | 8.3 (6.6 – 11.3) | 7.8 (6.5 – 10.6) | 0.68 |
| Group 3 (TU) | 7.7 (6.4 – 9.7) | 8 (6.4 – 10.3) | 0.75 |
| Group 4 (Training) | 8.3 (6.4 – 9.98) | 7.7 (6.4 – 10.3) | 0.08 |
| **Mobility-Tiredness Scale** |  |  |  |
| Group 1 (Combo) | 0 (0 – 1) | 0 (0 – 0) | **0.037** |
| Group 2 (Control) | 0 (0 – 1.25) | 0 (0 – 1) | 1.0 |
| Group 3 (TU) | 0 (0 – 1) | 0 (0 – 1) | 0.67 |
| Group 4 (Training) | 0 (0 – 2) | 0 (0 – 1) | 0.10 |
| **3 kg Arm Curls** |  |  |  |
| Group 1 (Combo) | 18 (15 – 20) | 17 (15 - 20) | 0.14 |
| Group 2 (Control) | 17 (15 – 20) | 17 (15 – 21) | 0.55 |
| Group 3 (TU) | 17 (13 – 21) | 17 (15 – 20) | 0.16 |
| Group 4 (Training) | 16 (13 – 19) | 17 (14 – 19) | **0.02** |
| **Montreal Cognitive Assessment** |  |  |  |
| Group 1 (Combo) | 24 (22 – 27) | 25 (24 – 27) | **0.038** |
| Group 2 (Control) | 25 (22 – 27) | 27 (24 – 28) | **0.0029** |
| Group 3 (TU) | 25 (24 – 26) | 26 (24 – 28) | **0.026** |
| Group 4 (Training) | 25 (23 – 27) | 26 (23 – 27) | 0.80 |
| **EQ-5D** |  |  |  |
| Group 1 (Combo) | 0.8 (0.78 – 0.86) | 0.86 (0.8 – 1) | **0.0007** |
| Group 2 (Control) | 0.8 (0.73 – 0.86) | 0.8 (0.72 – 0.86) | 0.62 |
| Group 3 (TU) | 0.79 (0.74 – 1) | 0.81 (0.74 – 1) | 0.41 |
| Group 4 (Training) | 0.78 (0.65 – 0.8) | 0.8 (0.72 – 0.86) | 0.39 |
| **EQ-5D Visual Analogue Scale** |  |  |  |
| Group 1 (Combo) | 80 (70 – 90) | 85 (80 – 90) | **0.023** |
| Group 2 (Control) | 75 (60 – 80) | 80 (65 – 90) | **0.028** |
| Group 3 (TU) | 75 (60 – 88) | 80 (63 – 86) | 0.52 |
| Group 4 (Training) | 78 (63 – 85) | 80 (63 – 86) | 0.40 |
| **Heart Rate Variability – SD2** |  |  |  |
| Group 1 (Combo) | 32 (26 – 47) | 53 (32 – 91) | **0.023** |
| Group 2 (Control) | 29 (20 – 39) | 34 (24 – 44) | 0.71 |
| Group 3 (TU) | 36 (24 – 46) | 32 (23 – 48) | 0.78 |
| Group 4 (Training) | 38 (23 – 66) | 33 (23 – 47) | 0.76 |

**Table S1. Changes within a group from baseline to week 20.** Median values are shown with 25 and 75 percentiles in brackets. *p<0.05 and **p<0.01 comparing baseline and week 20 results (Wilcoxon statistics). Endpoints where no group achieved statistically significant changes are not included. All statistically significant changes indicated improvements.

**Table S2**

**Blood tests with statistically significant changes within a group from baseline to week 20**

|  | **Baseline** | **Week 20** | **p-value** |
| --- | --- | --- | --- |
| **Hematocrit level (%)** |  |  |  |
| Group 1 (Combo) | 42 (40 – 45) | 45 (43 – 49) | **0.0001** |
| Group 2 (Control) | 43 (41 – 45) | 43 (40 – 44) | 0.60 |
| Group 3 (TU alone) | 42 (41 – 45) | 46 (43 – 49) | **<0.0001** |
| Group 4 (Training alone) | 43 (40 – 46) | 43 (41 – 45) | 0.35 |
| **Hemoglobin (mmol/L)** |  |  |  |
| Group 1 (Combo) | 8.8 (8.5 – 9.2) | 9.3 (8.9 – 9.9) | **0.0003** |
| Group 2 (Control) | 9 (8.2 – 9.5) | 8.9 (8.3 – 9.4) | 0.47 |
| Group 3 (TU) | 9.1 (8.5 – 9.4) | 9.6 (8.8 – 10) | **0.0002** |
| Group 4 (Training) | 8.9 (8.4 – 9.5) | 9 (8.5 – 9.4) | 0.83 |
| **Creatinine (μmol/L)** |  |  |  |
| Group 1 (Combo) | 75 (70 – 90) | 83 (72 – 96) | **<0.0001** |
| Group 2 (Control) | 83 (72 – 95) | 81 (73 – 97) | 0.97 |
| Group 3 (TU) | 85 (72 – 101) | 89 (80 – 97) | 0.14 |
| Group 4 (Training) | 85 (74 – 97) | 85 (76 – 100) | 0.54 |
| **Glucose (mmol/L)** |  |  |  |
| Group 1 (Combo) | 6 (5.6 – 6.5) | 6.4 (5.2 – 7.4) | 0.27 |
| Group 2 (Control) | 6.2 (5.7 – 7.6) | 6.6 (6 – 8.1) | **0.04** |
| Group 3 (TU) | 6.2 (5.4 – 6.9) | 6.1 (5.3 – 7.9) | 0.35 |
| Group 4 (Training) | 6 (5.5 – 7) | 6 (5.6 – 7) | 0.77 |
| **Total Cholesterol (mmol/L)** |  |  |  |
| Group 1 (Combo) | 4.6 (3.6 – 5) | 4.2 (3.4 – 4.9) | **0.003** |
| Group 2 (Control) | 4.3 (3.5 – 5) | 4.4 (3.7 – 4.9) | 0.83 |
| Group 3 (TU) | 4.2 (3.9 – 5.2) | 4.1 (3.4 – 5) | **0.0004** |
| Group 4 (Training) | 4.2 (3.3 – 5.1) | 4.3 (3.4 – 5.3) | 0.61 |
| **HDL (mmol/L)** |  |  |  |
| Group 1 (Combo) | 1.3 (1.1 – 1.5) | 1.2 (1 – 1.4) | **0.01** |
| Group 2 (Control) | 1.2 (1.1 – 1.6) | 1.3 (1.1 – 1.5) | 0.76 |
| Group 3 (TU) | 1.3 (1 – 1.6) | 1.2 (1 – 1.3) | **0.0002** |
| Group 4 (Training) | 1.3 (1.1 – 1.4) | 1.2 (1 – 1.3) | 0.053 |
| **LDL (mmol/L)** |  |  |  |
| Group 1 (Combo) | 2.3 (1.7 – 3.2) | 2 (1.6 – 2.7) | **0.009** |
| Group 2 (Control) | 2.2 (1.6 – 2.8) | 2.1 (1.6 – 2.7) | 0.52 |
| Group 3 (TU) | 2.3 (1.7 – 3.1) | 2.3 (1.7 – 2.8) | 0.08 |
| Group 4 (Training) | 2.4 (1.4 – 3.2) | 2.3 (1.6 – 3.1) | 0.81 |
| **Follitropin, FSH (IU/L)** |  |  |  |
| Group 1 (Combo) | 9.5 (6.1 – 27) | **1.8 (0.4 – 5.5)** | **<0.0001** |
| Group 2 (Control) | 9.3 (7 – 20) | 10 (7 – 20) | 0.82 |
| Group 3 (TU) | 9.4 (6.2 – 21) | **1.3 (0.3 – 4)** | **<0.0001** |
| Group 4 (Training) | 11 (6.9 – 44) | 10 (6.6 – 38) | 0.62 |
| **Lutropin, LH (IU/L)** |  |  |  |
| Group 1 (Combo) | 6.8 (3.7 – 13) | 0.3 (0.1 – 1.6) | **<0.0001** |
| Group 2 (Control) | 5.2 (4.2 – 7.7) | 5.1 (4.1 – 7) | 0.52 |
| Group 3 (TU) | 5.9 (4.2 – 9.2) | 0.3 (0.2 – 0.7) | **<0.0001** |
| Group 4 (Training) | 6.2 (4 – 14) | 7 (4 – 17) | 0.25 |
| **PSA (ng/ml)** |  |  |  |
| Group 1 (Combo) | 2 (0.9 – 2.8) | 2.5 (1.2 – 5.2) | **<0.0001** |
| Group 2 (Control) | 1.7 (0.4 – 2.7) | 1.7 (0.5 – 2.9) | 0.08 |
| Group 3 (TU) | 1 (0.6 – 2.4) | 1.2 (0.8 – 4) | **<0.0001** |
| Group 4 (Training) | 1.5 (0.7 – 3.9) | 1.5 (0.7 – 3.2) | 0.80 |
| **Vitamin D, 25(OH)D (nmol/L)** |  |  |  |
| Group 1 (Combo) | 81 (63 – 96) | 89 (75 – 111) | **0.007** |
| Group 2 (Control) | 70 (63 – 98) | 74 (56 – 90) | 0.89 |
| Group 3 (TU) | 83 (67 – 100) | 94 (71 – 106) | **0.02** |
| Group 4 (Training) | 88 (62 – 101) | 92 (75 – 114) | 0.08 |

**Table S2. Blood test changes within a group from baseline to week 20.** Median values are shown with 25 and 75 percentiles in brackets for comparing baseline and week 20 results (Wilcoxon statistics). Blood test parameters where no group achieved statistically significant changes are not included.

**Table S3**

**Bone Densitometry (DXA) with statistically significant changes within a group from baseline to week 20**

|  | **Baseline** | **Week 20** | **p-value** |
| --- | --- | --- | --- |
| **Weight** |  |  |  |
| Group 3 (TU) | 86 (77 – 100) | 84 (74 – 98) | **0.001** |
| **T-Score** |  |  |  |
| Group 4 (Training) | 0.8 (-0.1 – 1.4) | 0.8 (0 – 1.6) | **0.04** |
| **Z-Score** |  |  |  |
| Group 1 (Combo) | 0.9 (0.1 – 1.4) | 1.1 (0.3 – 1.5) | **0.004** |
| Group 4 (Training) | 1.3 (0.7 – 1.9) | 1.5 (0.9 – 2.4) | **0.02** |
| **Fat (kg)** |  |  |  |
| Group 1 (Combo) | 30 (23 – 35) | 28 (22 – 32) | **<0.0001** |
| Group 3 (TU) | 29 (23 – 34) | 27 (19 – 32) | **<0.0001** |
| **Fat free mass (kg)** |  |  |  |
| Group 1 (Combo) | 55 (50 – 61) | 58 (51 – 62) | **<0.0001** |
| **BMC (kg)** |  |  |  |
| Group 1 (Combo) | 2.9 (2.6 – 3.2) | 2.9 (2.6 – 3.3) | **0.03** |
| Group 3 (TU) | 3.1 (2.7 – 3.6) | 3.2 (2.8 – 3.6) | **0.02** |
| **Android fat (%)** |  |  |  |
| Group 1 (Combo) | 42 (37 – 51) | 43 (35 – 48) | **0.0001** |
| Group 3 (TU) | 41 (35 – 49) | 39 (31 – 47) | **0.0001** |
| **Gynoid fat (%)** |  |  |  |
| Group 1 (Combo) | 34 (29 – 36) | 31 (27 – 34) | **<0.0002** |
| Group 3 (TU) | 31 (29 – 35) | 29 (26 – 33) | **<0.0001** |
| **Legs fat mass (kg)** |  |  |  |
| Group 1 (Combo) | 5.7 (4.6 – 6.7) | 5.4 (4.3 – 6.1) | **<0.0001** |
| Group 3 (TU) | 5.9 (4.8 – 7.2) | 5.2 (4.3 – 6.9) | **<0.0001** |
| **Legs fat free mass (kg)** |  |  |  |
| Group 1 (Combo) | 17 (14 – 18) | 17 (14 – 19) | **0.003** |
| **Legs fat mass (%)** |  |  |  |
| Group 1 (Combo) | 36 (29 – 39) | 32 (26 – 37) | **<0.0001** |
| Group 3 (TU) | 35 (28 – 43) | 33 (27 – 39) | **<0.0001** |

**Table S3. Bone Densitometry changes within a group from baseline to week 20.** Median values are shown with 25 and 75 percentiles in brackets for comparing baseline and week 20 results (Wilcoxon statistics). BMC = Total Bone Mass. Parameters where no group achieved statistically significant changes are not included in the table. Also due to the large amount of parameters, only results from groups showing statistically significant changes have been included. The control group achieved no statistically significant changes from baseline to week 20.

**Supplementary references**

S1. Avlund K, Thudium D, Davidsen M, Fuglsang-Sørensen B. Are selfrating functional ability reliable? *Scandinavian Journal of Occupational Therapy.* 1995;**2**:10–6.

S2. [Bech P](https://www.ncbi.nlm.nih.gov/pubmed/?term=Bech%20P%5BAuthor%5D&cauthor=true&cauthor_uid=11578668), [Rasmussen NA](https://www.ncbi.nlm.nih.gov/pubmed/?term=Rasmussen%20NA%5BAuthor%5D&cauthor=true&cauthor_uid=11578668), [Olsen LR](https://www.ncbi.nlm.nih.gov/pubmed/?term=Olsen%20LR%5BAuthor%5D&cauthor=true&cauthor_uid=11578668), [Noerholm V](https://www.ncbi.nlm.nih.gov/pubmed/?term=Noerholm%20V%5BAuthor%5D&cauthor=true&cauthor_uid=11578668), [Abildgaard W](https://www.ncbi.nlm.nih.gov/pubmed/?term=Abildgaard%20W%5BAuthor%5D&cauthor=true&cauthor_uid=11578668). The sensitivity and specificity of the Major Depression Inventory, using the Present State Examination as the index of diagnostic validity. [*J Affect Disord.*](https://www.ncbi.nlm.nih.gov/pubmed/11578668) 2001 Oct;66(2-3):159-64.

S3. Nasreddine ZS, Phillips NA, Bédirian V, Charbonneau S, Whitehead V, Collin I, et al. The Montreal Cognitive Assessment, MoCA: a brief screening tool for mild cognitive impairment*. J Am Geriatr Soc.* 2005;53:695–699.

S4. Rabin R and de Charro F. EQ-5D: a measure of health status from the EuroQol Group. Ann Med 2001; 33: 337–343.

S5. Lerdal A, Kottorp A, Gay C, Aouizerat BE, Portillo CJ, Lee KA. A 7-item version of the fatigue severity scale has better psychometric properties among HIV-infected adults: an application of a Rasch model. *Qual Life Res*. 2011 Nov;20(9):1447-56.

S6. Rogers RG, Coates KW, Kammerer-Doak D, Khalsa S, Qualls C. A short form of the Pelvic Organ Prolapse/Urinary Incontinence Sexual Questionnaire (PISQ-12). Int Urogynecol J Pelvic Floor Dysfunct. 2003 Aug;14(3):164-8; discussion 168.
